# Regulatory Specialization of Xyloglucan (XG) and Glucuronoarabinoxylan (GAX) in Pericarp Cell Walls during Fruit Ripening in Tomato (*Solanum lycopersicum*)

**DOI:** 10.1371/journal.pone.0089871

**Published:** 2014-02-26

**Authors:** Ayami Takizawa, Hiromi Hyodo, Kanako Wada, Tadashi Ishii, Shinobu Satoh, Hiroaki Iwai

**Affiliations:** University of Tsukuba, Faculty of Life and Environmental Sciences, Tsukuba, Ibaraki, Japan; Iowa State University, United States of America

## Abstract

Disassembly of cell wall polysaccharides by various cell wall hydrolases during fruit softening causes structural changes in hemicellulose and pectin that affect the physical properties and softening of tomato fruit. In a previous study, we showed that the changes in pectin during tomato fruit ripening were unique in each fruit tissue. In this study, to clarify the changes in hemicellulose in tissues during tomato fruit ripening, we focused on glucuronoarabinoxylan (GAX) and xyloglucan (XG). GAX was detected only in the skin and inner epidermis of the pericarp using LM11 antibodies, whereas a large increase in XG was detected in all fruit tissues using LM15 antibodies. The activity of hemicellulose degradation enzymes, such as β-xylosidase and α-arabinofuranosidase, decreased gradually during fruit ripening, although the tomato fruits continued to soften. In contrast, GAX and XG biosynthesis-related genes were expressed in all tomato fruit tissues even during ripening, indicating that XG was synthesized throughout the fruit and that GAX may be synthesized only in the vascular bundles and the inner epidermis. Our results suggest that changes in the cell wall architecture and tissue-specific distribution of XG and GAX might be required for the regulation of fruit softening and the maintenance of fruit shape.

## Introduction

Fruit ripening and softening are major factors affecting the perishability of fleshy or climacteric fruits. Fleshy fruits soften during ripening mainly as a consequence of the disassembly of different cell-wall components. Depolymerization and solubilization of pectic and hemicellulosic polysaccharides during softening have been reported [1]–[5].

The extent of cell wall modification and which modifying enzymes are active during fruit softening depends on the fruit species. The cell wall polysaccharide composition of the fruit also differs between fruit species. Tomato has been used as a model system for intensive study of ripening and softening [6] [7] [8] [9], but the molecular mechanisms of fruit softening are still not completely understood.

Changes in cell wall degradation and biosynthesis and cross-linkage of cell wall polysaccharides which play a role in fruit softening and fruit shape maintenance during fruit ripening might differ between fruit tissues. Therefore, we focused on glucuronoarabinoxylan (GAX) and xyloglucan (XG) cell wall matrix polysaccharides that are thought to be cross-linked to other cell wall polysaccharides.

XG is the most abundant hemicellulose in the primary cell walls of non-graminaceous plants, where it coats and cross-links adjacent cellulose microfibrils through non-covalent associations [10]–[13]. XG degradation is a central factor in models of wall modification that occurs during transient wall loosening in expanding cells or in terminal wall degradation during fruit ripening and organ abscission [13]–[15]. XG endotransglycosylase/hydrolase (XTH) enzymes play a key role in fruit ripening by loosening the cell wall, which increases the accessibility of the cell wall to other cell wall-associated enzymes. The pattern of XG-degrading enzyme activity in ripening tomato fruit is apparently complex [16] and may reflect a combination of hydrolases, transglucosylases, and/or enzymes with both activities.

GAX is a major hemicellulose in the secondary cell walls of dicots and all cell walls of grass species [17]. Most xylans consist of β-d-xylopyranosyl residues that form a core backbone, which may be substituted with α-l-arabinofuranosyl (arabinoxylans), and to a lesser extent, α-d-glucuronic acid (glucuronarabinoxylan) residues. The cell walls of the inner and outer pericarp of tomato fruits contain arabinose and xylose as prominent components [18], the latter including XGs [2]. The chemical structures of wall XG and GAX are subject to modification during plant growth and development, including during seed germination, fruit development, and ripening and abscission [19]–[23]. α-l-Arabinofuranosidase (EC 3.2.1.55) and β-d-xylosidase (EC 3.2.1.37) are responsible for the hydrolysis of XG and GAX liberating α-l-arabinofuranosyl residues and β-d-xylosyl residues, respectively. Β-d-Xylosidase and α-l-arabinofuranosidase have recently been identified in developing and ripening tomato fruits [24]. The activity of both enzymes was highest during early fruit growth, before decreasing during later development and ripening [24].

Several genes from *Arabidopsis thaliana* (Arabidopsis), poplar, and some other plants were shown to be associated with GAX biosynthesis. In the Arabidopsis genome, four glycosyltransferases from the GT43 family, IRX9/I9H and IRX14/I14H, were shown to be required for the normal elongation of the GAX backbone [25] [26] [27] [28] [29] [30].

In a previous study, we showed that the changes in pectin during tomato fruit ripening were unique in each fruit tissue. In this study, to understand the changes of hemicellulose in tissues during tomato fruit ripening, we examined the gene expression and the enzymatic activities involved in GAX and XG synthesis and degradation in each fruit tissue. We also analyzed the monosaccharide compositions of GAX and XG and determined their distribution in fruit tissues by immunohistochemical analysis.

## Materials and Methods

### Plant material

**Figure 1:**
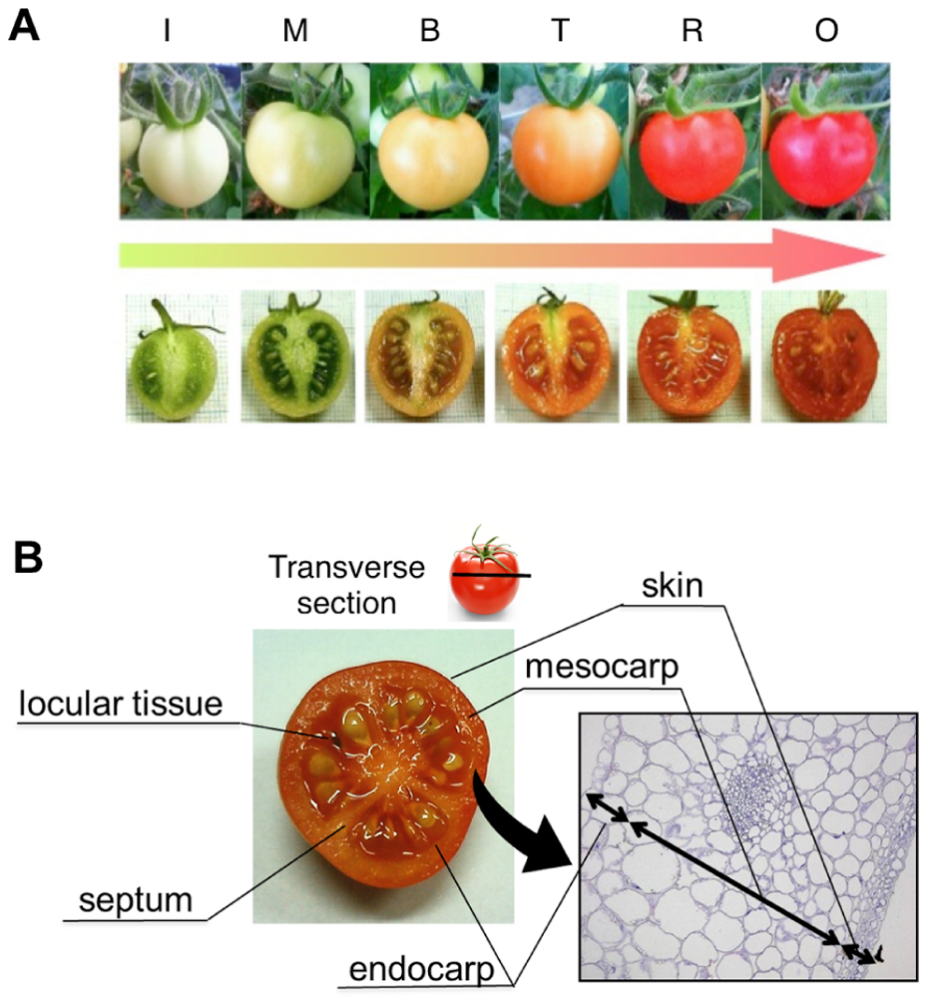
Preparation for tissue-specific analysis. A, The fruit ripening stages of cv. Micro Tom. The six stages included immature green (I), mature green (M), breaker (B), turning (T), red ripe (R), and overripe (O). B, The fruit tissues of cv. Micro Tom. The eight tissues included skin, mesocarp, endocarp, septum, locular tissue, seed, placenta. and core. These were separated by hand-sectioning.

Tomatoes (*Solanum lycopersicum* cv. ‘Micro Tom’) were grown inside a cultivation chamber (TOMY CL-301) under 16-h light and 8-h dark at 26°C and 22°C, respectively, and a light intensity of approximately 100 µmol m^−2^ s^−1^. Tomato fruits at the corresponding developmental stages were also collected: I [<1 cm length; 15 days after pollination (dap)], M (30 dap), B (35 dap), T (37 dap), R (45 dap), and OR (55 dap); Fig. 1).

### β-D-xylosidase and α-L-arabinofuranosidase assay

Enzymes were extracted in 20 mM sodium acetate buffer (pH 5.0) with 1 M NaCl [31]. Each tissue (0.3 g) was ground in liquid nitrogen, extracted in sodium acetate buffer, and then centrifuged at 15,000× g for 30 min at 4°C. Hydrolytic activities of the cell wall proteins extracted from each tissue toward synthetic substrates were determined using a reaction mixture (200 µl) consisting of the cell wall proteins, 25 mM acetate buffer (pH 5.0), and 1 mM PNP-glycoside substrate. After incubation at 37°C for 2 h, the reaction was terminated by the addition of 200 mM sodium carbonate (800 µl) and absorbance was monitored at 420 nm. One unit of enzyme activity liberates 1 µmol of *p*-nitrophenol min^−1^.

### RNA expression analysis

Total RNA extractions were performed using a Qiagen RNeasy Mini Kit (Qiagen, Valencia, CA, USA) with subsequent DNase treatment to remove any contaminating DNA. RNA was quantified by spectroscopy. Quantitative reverse transcription-polymerase chain reaction (RT-PCR) assays were performed using an ExTaq Kit (TaKaRa Bio, Otsu, Japan). Degenerate primers were synthesized based on conserved sequence regions obtained by searching the SOL database (http://solgenomics.net/) and Mibase (http://www.pgb.kazusa.or.jp/mibase/). For SlIRX9-L1, the upstream primer was 5′-TTCTTGCTCATTCAGGTGGTGTGG-3′ and the downstream primer was 5′-TACTTGTTCTCCGCTGATTGCCTG-3′. For SlIRX9-L2, the upstream primer was 5′-ATCCCATACGACAGTTGGAC-3′ and the downstream primer was 5′-CTTCTGTGATGGCAGTAGCA-3′. For XST1, the upstream primer was 5′-GACTCAATCGTACCGGAGAATC-3′ and the downstream primer was 5′-GGCTAGAACGATTACGTGACCT-3′. For XST2, the upstream primer was 5′-AGCTCAAACACGCTTTCCAC-3′ and the downstream primer was 5′-GCTGCTCTTTAAGCCAATCG-3′. For SlXXT-L, the upstream primer was 5′-TGATGAGGATGATGGTGAGC-3′ and the downstream primer was 5′-CCCTTACCTTTCCCTTTGGT-3′. RT-PCR conditions for the SlURX9-L1 and SlURX9-L2 RNAs were 30 cycles at 94°C for 30 sec, 51°C for 30 sec, and 72°C for 1 min. The conditions for PCR of the SlXST1 and SlXST2 RNAs were identical, except that the annealing temperature was 58°C with 35 cycles and 30 cycles, respectively. The conditions for PCR of the SlXXT-L RNA were identical except that the annealing temperature was 53°C with 20 cycles. The RNA expression levels were visualized on ethidium bromide-stained gels.

### Extraction and analysis of cell wall polysaccharides

Tomato skin, mesocarp/endocarp, septum, locular tissue, and seed tissue samples (300 mg) were extracted and frozen in liquid nitrogen. The frozen tissue was ground to a powder in a mortar in liquid nitrogen and the resulting powder was washed in 80% ethanol (EtOH). The supernatant was removed after centrifugation for 5 min at 15,000× g. The pellet was washed three times with water, three times with 1 ml methanol:chloroform (MC, 1∶1), and three times with 1-ml acetone. One milliliter of phenol:acetic acid:water (PAW, 2∶1∶1) was added to the pellet and mixed. MC (1 ml) was added to the sample and then washed with 1 ml of acetone. The samples were then air-dried for more than 12 h. Alcohol-insoluble residue (AIR) was used as the cell wall material. A total of 2 mg of AIR was boiled with 0.25% ammonium oxalate for 2 h. After boiling, the samples were centrifuged at 15,000× g for 5 min. The supernatant was the ammonium oxalate-soluble fraction. The pellets were hydrolyzed with 2 M trifluoroacetic acid (TFA) at 121°C for 2 h. After hydrolysis, the samples were centrifuged at 15,000× g for 5 min. The supernatant was taken as the TFA-soluble fraction representing a hemicellulosic sugar fraction. The pellets were hydrolyzed with 72% sulfuric acid (H_2_SO_4_) at room temperature for 2 h and then diluted to 4% H_2_SO_4_ and boiled for 1 h. The H_2_SO_4_ solutions were neutralized with barium hydroxide. Sugars in the TFA-soluble fractions were treated with methanol-hydrogen chloride and then converted to trimethylsilyl derivatives and analyzed by gas-liquid chromatography (GC-2010; Shimadzu, Kyoto, Japan). Sugar contents of the TFA-soluble fractions were determined using the phenol-H_2_SO_4_ method.

### Immunohistochemical Analysis

Tomato fruit samples were cut in half by hand-sectioning. The samples were fixed in 2.5% paraformaldehyde in 0.025 mM PBS and evacuated with a vacuum pump for 24 h. Fixed samples were dehydrated through the following series of EtOH concentrations: 30%, 50%, 70%, 80%, 90%, and 95% three times for 20 min each and then 100% three times for 30 min each. EtOH in dehydrated samples was exchanged for Technovit 7100 resin through the following series of Technovit 7100 in EtOH: 50% for 6 h, then 100% for 6 h and 12 h. Samples were then solidified in Technovit 7100 resin following the manufacturer's protocol. Embedded samples were cut into 10-µm sections using a microtome and a tungsten knife.

A series of monoclonal rat IgG antibodies was purchased from PlantProbes (Leeds, UK; www.plantprobes.net), anti-XTH rabbit antibody was obtained from Agrisera (Vännäs, Sweden; www.agrisera.com/en/index.html), and a TSA kit with horseradish peroxidase (HRP)-conjugated secondary antibody and Alexa Fluor 488 tyramide were acquired from Invitrogen (Carlsbad, CA, USA; cat. no. T20912). Immunohistochemistry using the set of monoclonal antibodies followed the manufacturer's instructions. The sections were put under PBS prior to labeling and 100 µl of the following reagents were dropped onto the sections in the following order: quenching buffer (to quench endogenous peroxidase activity), 1% blocking reagent, and primary antibody diluted in 1% blocking reagent (1∶30). The sections were incubated each time at room temperature for 1 h. The sections were washed three times with PBS, then incubated in 100 µl of HRP conjugate diluted in 1% blocking reagent (1∶100) for 1 h, washed (3×PBS), and incubated in 100 µl of tyramide working solution (tyramide stock solution diluted in amplification buffer/0.0015% hydrogen peroxide (H_2_O_2_); 1∶100) for 10 min at room temperature and washed three times with PBS followed by distilled water (DW) twice. The sections were mounted in DW and observed under a fluorescence microscope.

### Toluidine Blue Dye Penetration Test

The method for examination of cuticular integrity was adapted from Tanaka et al. [32]. An aqueous solution of 0.01% (w/v) Toluidine Blue O (Sigma-Aldrich, St. Louis, MO, USA) was poured into the fruit inner locular cavity, after the seeds were removed, until completely filled. In fruit with two or more locules, one of the locules was used for the test and the other one was filled with water as the negative control. After 2 min, the Toluidine Blue O solution was removed, and the inner surface of the locule was rinsed with water to remove excess Toluidine Blue O solution. Small holes in the surface were produced with a pin to allow penetration of the dye in discrete areas as the positive control.

## Results

### Determination of XG and GAX Degradation-Related Enzyme Activity in Tomato Fruit Tissues during Ripening

α-l-Arabinofuranosidase (EC 3.2.1.55) and β-d-xylosidase (EC 3.2.1.37) are responsible for the hydrolysis of arabinoxylans, xylans, and xyloglucans liberating α-l-arabinofuranosyl residues and β-d-xylosyl residues, respectively. Little is known about the biochemical properties of these enzymes during fruit ripening, although a xylosidase-like gene was recently identified in ripening Japanese pear [33]. In tomato fruit, β-d-xylosidase and α-l-arabinofuranosidase are reported here; the presence of three isoforms of the latter during tomato fruit development and ripening was reported [22].

**Figure 2:**
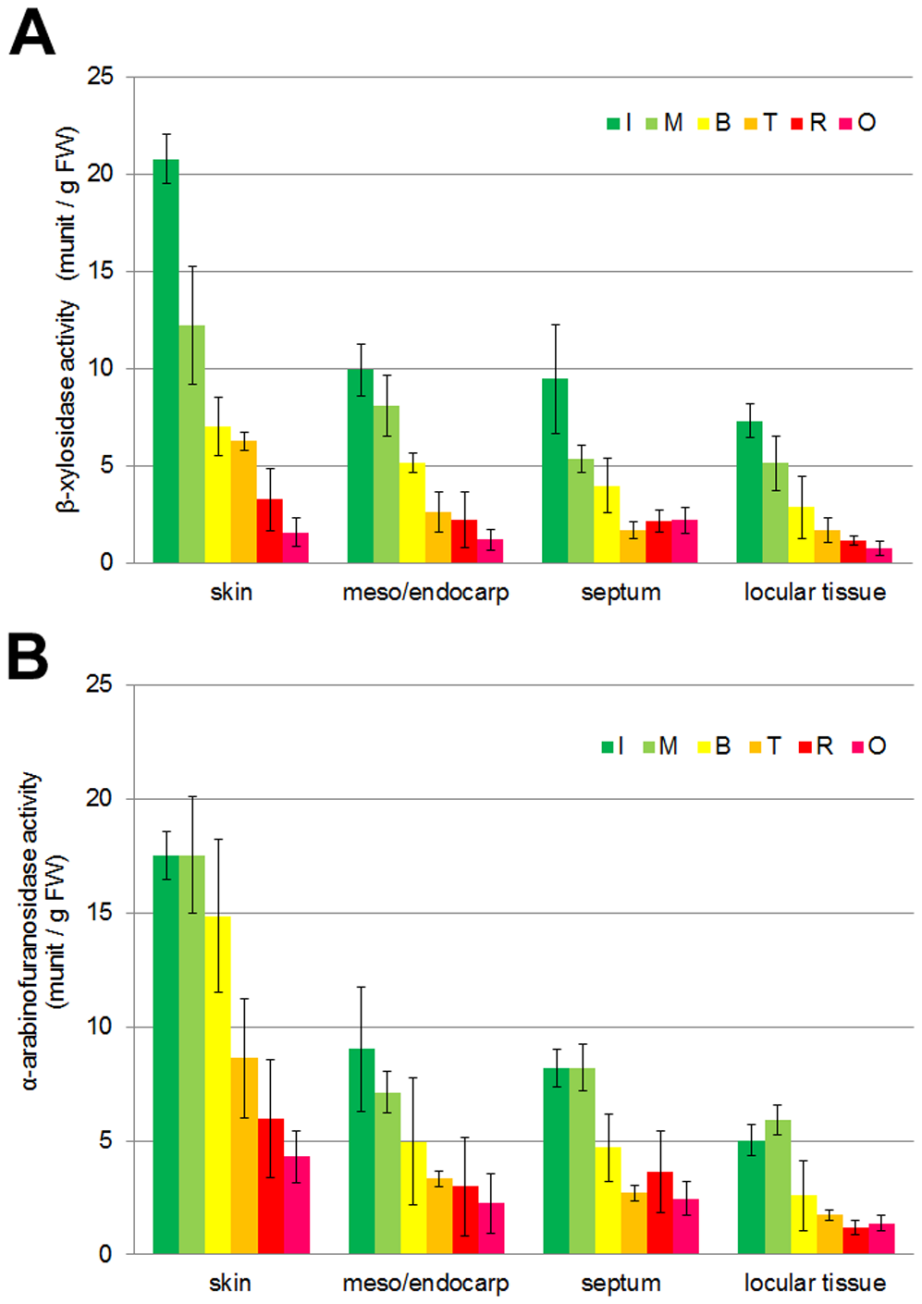
β-Xylosidase and α-arabinofuranosidase activities gradually decreased in fruit tissues during ripening. A, β-Xylosidase activity. B, α-Arabinofuranosidase activity. Total protein in the cell wall was extracted from each fruit tissue and assayed for β-xylosidase and α-arabinofuranosidase activity. The five tissues analyzed in these assays included skin, mesocarp/endocarp, septum, locular tissue, and seed. Ripening stage: I, immature green; M, mature green; B, breaker; T, turning; R, red ripe; O, overripe. ±SD of three independent replicates.

The activities of both enzymes were assayed in extracts from tomato fruit skin, mesocarp/endocarp, septum, locular tissue, and seeds [31]. Both enzyme activities were high during the immature green stage, and decreased gradually during the fruit ripening stage (Fig. 2). Although α-l-arabinofuranosidase activity was similarly high in both the immature green and mature green stage (Fig. 2B), β-d-xylosidase activity was remarkably high in the immature green stage (Fig. 2A). These data were based on fresh weight, and the protein-based data were very similar in the two tissues (Supporting Information Fig. S1 in File S1).

### Analysis of XG and GAX Biosynthesis-Related Gene Expression in Tomato Fruit Tissues during Ripening

**Figure 3:**
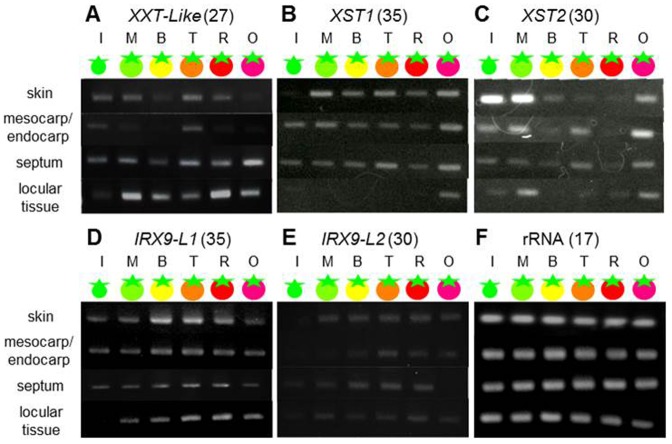
XG and GAX biosynthesis-related gene expression patterns differed among tissues. Gene expression was analyzed by RT-PCR. A, α-1,6-xylosyltransferase involved in XG biosynthesis, SlXXT-L: SGN-U581426 (27 cycles); B, arabinosyltransferase involved in XG biosynthesis, SlXST1: Sl07g044960 (35 cycles); C, SlXST2: Sl07g049610 (30 cycles), D, β-1,4-xylosyltransferase involved in GAX biosynthesis, SlIRX9-L1: SGN-U583344 (35 cycles); E, SlIRX9-L2: SGN-U568972 (30 cycles); D, 17S rRNA, as a control (17 cycles). Expression levels were compared to *rRNA* in the same assay. The four tissues analyzed in these assays included skin, mesocarp/endocarp, septum, and locular tissue. Ripening stages were as follows: I, immature green; M, mature green; B, breaker; T, turning; R, red ripe; O, overripe.

To compare ripening-related cell-wall XG and GAX metabolism between tomato fruit tissues, the expression levels of genes encoding proteins involved in XG and GAX biosynthesis, including α-1,6-xylosyltransferase (SlXXT-L: SGN-U581426), arabinosyltransferase (SlXST1: Sl07g044960, SlXST2: Sl07g049610), and β-1,4-xylosyltransferase (SlIRX9-L1: SGN-U583344, SlIRX9-L2: SGN-U568972), were examined by RT-PCR (Fig. 3). The XXT1-5 genes were identified in Arabidopsis as XG biosynthesis-related genes belonging to the CAZy glycosyltransferase 34 (GT34) family. The XXT1–5 genes, especially XXT1, XXT2, and XXT5, have been shown biochemically to be responsible for α-1,6-xylosyltransferase activity [34] [35] [36]. An α-1,6-xylosyltransferase gene had not been previously identified in tomato, so we examined genes homologous to XXT1, XXT2, and XXT5. Homology searches of the SOL database (http://solgenomics.net/) and Mibase (http://www.pgb.kazusa.or.jp/mibase/) identified a gene (SlXXT-L) that was homologous to XXT1, XXT2, and XXT5 and contained a conserved domain of the GT34 family in tomatoes. SlXXT-L gene expression increased in two steps. Expression was high during the mature green and overripe stages (Fig. 3C). In the ripening stage, this expression increased from the turning stage. Recently, two arabinosyltransferases related to XG metabolism were identified unambiguously in tomato [37]. We examined the tissue-specific expression of the genes encoding the SlXST1 and SlXST2 arabinosyltransferases as representative XG biosynthesis-related genes using RT-PCR analysis (Fig. 3). XST1 and XST2 expression increased in two steps corresponding to the fruit development and fruit ripening stages (Fig. 3C). The expression patterns of XST1 and XST2 were almost identical to those of SlXXT-L. A GAX biosynthesis-related gene, IRX9, was identified in Arabidopsis. The IRX9 gene encoded β-1,4-xylan elongating enzyme, a putative member of the glycosyltransferases 43 (GT43) family and was expressed coordinately with cellulose synthase subunits during secondary cell wall formation. Similar to XXT-L, we found two genes homologous to IRX9 (SlIRX9-L1, SlIRX9-L2) that included a conserved domain of the GT43 family in tomatoes. Expression of both genes increased in the early ripening stage and decreased at later stages. The peak of expression differed in each tissue. The peak for skin in the breaker stage was earlier than other tissues, and the peak in locular tissue was latest at the red ripe stage (Fig. 3A, B).

### Determination of xylose and arabinose contents in fruit tissues during ripening

**Figure 4:**
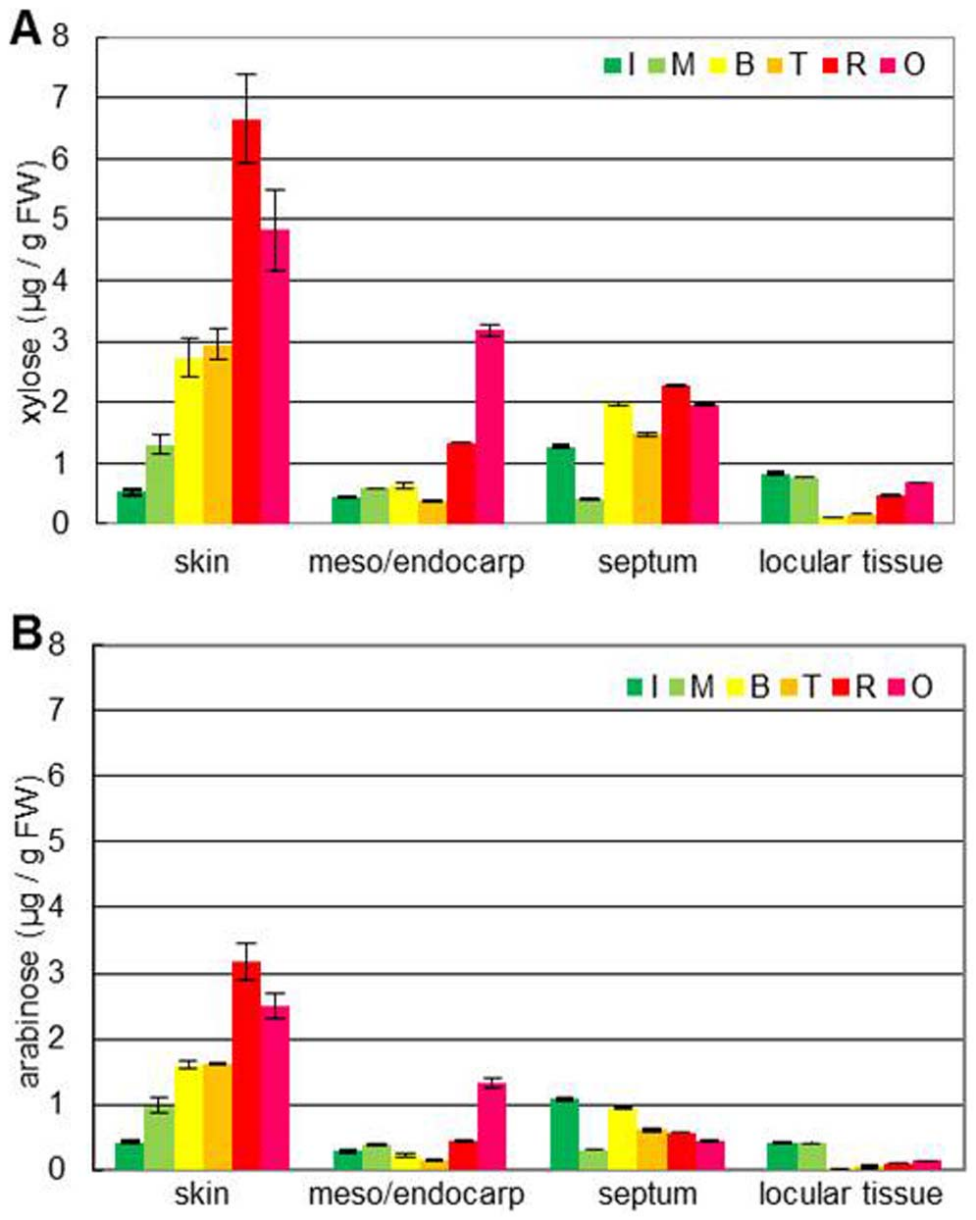
Changes in xylose and arabinose content differed in fruit tissues during ripening. A, Xylose content per 1/endocarp, septum, and locular tissue. Ripening stage: I, immature green; M, mature green; B, breaker; T, turning; R, red ripe; O, overripe. ±SD of three independent replicates.

Changes in the amounts of hemicellulosic sugars, xylose, and arabinose (on a fresh weight basis) followed typical ripening-related trends (Fig. 4A–4C). Xylose content increased during fruit ripening in the skin, mesocarp/endocarp, and septum. Glucose content also increased in these tissues during fruit ripening (Supporting Information Fig. S2 in File S1). In contrast, the xylose content in locular tissue decreased from the mature green to the turning stage and increased again during the red ripe stage (Fig. 4A). Changes in arabinose content were similar to xylose, although the total content was lower than that of xylose (Fig. 4B). All sugar levels were high in the skin and low in locular tissue.

### Distribution of XG and GAX in tomato fruit tissues during ripening

**Figure 5:**
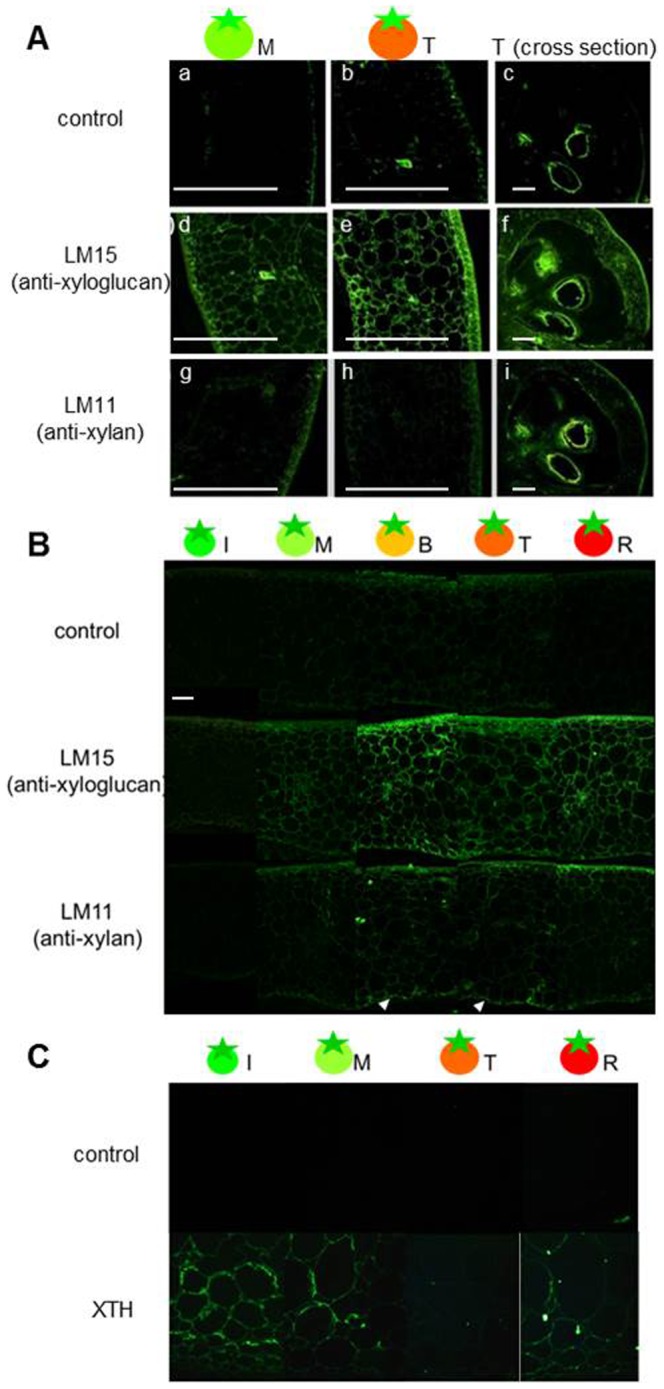
Immunolocalization of XG, GAX, and XTH epitopes in tomato fruit longitudinal and cross sections. A, Immunolocalization of XG and GAX in fruit sections. Top panels: Negative control (treated with only secondary antibodies). Middle panels: Xyloglucan immunolabeled with LM15 antibody. Bottom panels: Glucuronoarabinoxylan immunolabeled with LM11 antibody. Bars represent 1 mm. B, Immunolocalization of XG and GAX in pericarp. Top panels: Negative control. Middle panels: Immunolabeled with LM15 antibody. Bottom panels: Immunolabeled with LM11 antibody. Bars represent 0.2 mm. C, Immunolocalization of XTH in pericarp. Top panels: Negative control. Bottom panels; immunolabeled with anti-XTH antibody. Bars represent 0.2 mm. Ripening stage: M, mature green; T, turning; R, red ripe.

XG and GAX levels were determined at the cellular level using immunohistochemistry. LM15 monoclonal antibodies were raised against XG, and LM11 monoclonal antibodies were raised against GAX (http://www.plantprobes.net/index.php) (Fig. 5A, 5B). The LM15 signal increased overall during fruit during ripening. In contrast, the LM11 signal was localized to the skin and inner epidermal cells of the pericarp. Both signals in locular tissue were lower than in other tissues. Additionally, XTH was determined by immunolocalization using polyclonal antibodies (Fig. 5C). In tomato, the *SlXTH1-25* genes were reported to encode XTHs [38]. An XTH polyclonal antibody detected all of the XTH proteins. The XTH signal was high in the fruit developmental stage and decreased in the turning stage. The signal increased again in the red ripe stage (Fig. 5C).

### Distribution of the cuticle in the inner epidermis of tomato fruit

**Figure 6:**
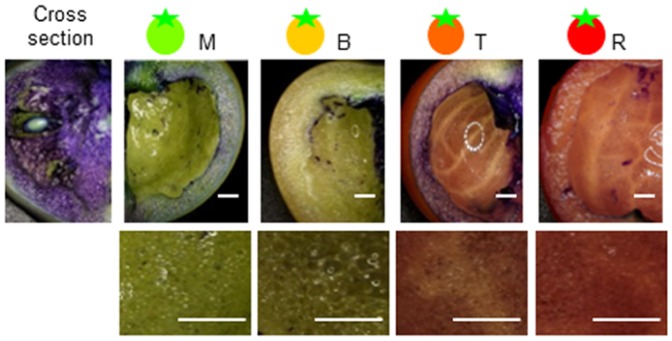
Inner surface of the inner fruit pericarp following Toluidine Blue dye staining. The permeability and integrity of the inner epidermal lipid membrane was tested with a Toluidine Blue O dye penetration test. The lower panels are shown at greater magnification. The test was applied to fruit at four ripening stages: MG, B, T, and R. Although the pericarp and locular tissues were stained, the inner epidermis of the pericarp was not stained in any stage. M, mature green; B, breaker; T, turning; R, red ripe.

GAX epitopes (LM11) were detected in the outer and inner epidermis (Fig. 5A, 5B). Recently, a cuticle lining was identified not only in the outer epidermis, but also in the inner epidermis of the tomato fruit pericarp [39]. However, recent investigations have not confirmed the presence of the cuticle lining in the inner epidermis of fruit during the ripening stage. To confirm the presence of the cuticle in the inner epidermis, we stained the inner tomato fruit with Toluidine Blue. The permeability and integrity of the inner epidermal lipid membrane were tested using a Toluidine Blue O dye penetration test [32]. Cell walls are stained by the dye if stomata, pores, or other apertures exist, but the dye does not penetrate hydrophobic membranes such as the cuticle. The test was applied to fruit at four ripening stages: M, B, T, and R. Although the pericarp and locular tissue were stained, the inner epidermis of the pericarp was not stained at any of the ripening stages. The inner surface of the pericarp was impermeable to the dye solution at all fruit stages and no staining was observed (Fig. 6). These results suggested that the cuticle was present in the inner epidermis of ripening-stage fruit.

## Discussion

### Hemicelluloses of the pericarp cell walls are specialized and reconstructed during fruit ripening

Tissue softening is one of the most important changes that occur during the development of a fleshy fruit. Fruit softening occurs during ripening as a consequence of a modification of the cell wall structure. The main changes observed are in pectic and hemicellulosic polysaccharides that undergo solubilization and depolymerization [1]–[3]. These changes in the cell wall structure, which lead to the loss of tissue integrity, are mediated by the action of specific cell wall hydrolases, such as pectinases and hemicellulases. In this study, β-xylosidase and α-arabinofuranosindase activities, which are associated with the degradation of GX and GAX, decreased during fruit ripening. In contrast, IRX9-like genes, with conserved domains of the glycosyltransferase family 43 members, which are proposed to function during GAX backbone elongation [25]–[30], were increasingly expressed during ripening and were especially active in the skin. Also, XXT-L genes encoding xylosyltransferases participating in XG biosynthesis [36] were expressed in two stages: the fruit development stage and later ripening stage. These results suggest that XG and GAX are not only degraded, but also synthesized during fruit ripening. In the results of the hemicellulosic sugar composition analysis, glucose, xylose, and arabinose increased during ripening, especially in pericarp tissues, such as the skin and mesocarp. In locular tissue, despite the high expression of XG and GAX synthesis-related enzyme genes, xylose and arabinose content decreased in the early ripening stage, although their content increased in the later ripening stage. In pericarp and locular tissues, although degradation enzyme activity similarly decreased, synthesis-related enzyme gene expression increased at different stages, earlier in pericarp tissue than in locular tissue. In our previous study, changes in pectin content and structure differed in these tissues, and hairy pectin increased particularly in the skin [40]. Similar to pectin, hemicellulosic sugar content, especially xylose and arabinose, increased in the skin. Cell wall composition and structure may become complex-specific in the skin. Even during the ripening stage, hemicellulosic polysaccharides such as XG and GAX may be synthesized as well as degraded with pectin and other cell wall components in each tissue.

### Distribution and metabolic pattern of XG and GAX differed during fruit ripening

In this study, we showed that biosynthesis of both XG and GAX occurred even during the fruit-ripening stage and that the distributions of these polysaccharides in fruit tissues differed. XG epitopes were generally detected by LM15 antibody in every fruit tissue and the levels of XTH epitope signals increased in two steps during the fruit development and ripening stages. In a previous study, XTH enzymes were found to play a key role in fruit ripening by loosening the cell wall in preparation for further modification by other cell-wall-associated enzymes and through the disassembly of XG. XTH can act as a transglucosylase (XET) with dual roles, integrating newly secreted XG chains into an existing wall-bound XG, restructuring the existing cell wall material by catalyzing transglycosylation between previously wall bound XG molecules, and acting as a hydrolase (XEH), hydrolyzing one XG molecule, depending on the nature of the XG donor and acceptor substrates [41]–[43]. XTH enzymes are thought to play a key role in fruit ripening by loosening the cell wall in preparation for further modification by other cell-wall-associated enzymes and through the disassembly of XG. In tomato fruits, SlXTH1 was found to be highly expressed during fruit development and *SlXTH5* was observed to have the highest expression of any *SlXTH* during ripening [6]. Additionally, these increases in expression could be contributing to the increase in XET-specific activity detected by ethylene treatment [39]. The results of our studies are consistent with previous studies showing that XG increased during fruit ripening. Therefore, in these genes, the two-step expression and two activity types, XEH and XET, contribute to cell wall reconstruction during fruit ripening.

In contrast, only a few GAX epitopes were detected by LM11, and these were localized in the outer border of the pericarp, such as in the skin and inner epidermis. GAX in dicots is found mainly in the vascular bundle, and our results suggest that GAX was localized even in fruit pericarp tissues. Additionally, GAX degradation-related enzyme activity gradually decreased during fruit ripening, and biosynthesis-related enzyme gene expression increased during ripening. Based on these results, GAX may be involved in tomato fruit body maintenance, especially in the cell layers surrounding the pericarp. The distribution of hemicellulose polysaccharides differed for XG and GAX, and cell wall reconstruction may occur in each tissue or cell layer.

### Role of hemicellulose for the maintenance of the cell wall network during fruit ripening

Various cell wall-related proteins involved in the reconstruction of cell walls remodel the chemical structure and interactions of cell wall pectin, hemicelluloses, and cellulose in the cell wall during fruit ripening and softening [45] [46]. Early models proposed that fruit softening occurs during ripening as a consequence of modification of the cell wall structure, and suggested that enzymes such as polygalacturonase (PG), which can hydrolyze the pectin backbone of HG polymers de-esterified by pectin methylesterase (PE), might play a major role in controlling texture changes in tomatoes [47] [48]. Pectin is a major component of the tomato fruit cell wall and substantial changes occur in the activity levels of these enzymes during the ripening process. However, experiments in which genes encoding these and other wall remodeling proteins have been silenced in transgenic tomato fruits have not supported this hypothesis. These experiments indicated that although small effects on fruit softening can be achieved by individual gene knockdown [49] [50], substantial changes in fruit texture are likely to require the simultaneous modulation of multiple genes.

Fruit hemicellulose is composed of XG, glucomannan (GM), and GAX [51]. XG and GM structures are rearranged by xyloglucan- or mannan-transglucosylase/hydrolases (XTH, MTH) during fruit development and ripening [6], [52]–[54] while expansin proteins modulate the hydrogen interaction of XG with cellulose [54], particularly during fruit softening [55]. Recently, a study reported that at the onset of ripening, a substantial decrease occurs in the expression of at least four cellulose synthase genes and also those encoding a variety of glycosyl hydrolases [56]. This is in contrast to genes annotated as XTHs [6], in which 10 members of this family show a ripening-related burst of expression. These new data suggest a more important role for members of the XTH family in ripening-associated cell wall changes than previously suspected [44]. In this study, the XG content increased in early fruit ripening stages and XTH epitopes increased in two steps during the mature green and turning stages consistent with a ripening-related burst of XTH family gene expression. These XTH genes may have hydrolase and endotransglucosylase activity. Like the XTH family, endo-β-mannanase was suggested to have both hydrolase and endotransglucosylase activity in tomato fruits, and mannans in tomato fruit may not be depolymerized during ripening despite the presence of endo-β-mannanase. Transglycosylation of hemicellulosic polysaccharides would remodel rather than weaken the cell wall and allow the fruit epidermis to possibly retain flexibility and plasticity to resist cracking and infection when the fruit is ripe. GAX also increased during fruit ripening, especially at the border of the fruit pericarp, such as in the skin and inner epidermis. In a previous study, we showed that the changes in pectin during tomato fruit ripening were unique in each fruit tissue. In particular, de-methylesterified pectin-Ca cross-linkages and hairy pectin regions increased in skin during fruit ripening [40]. Moreover, the cuticle layer was reported to include not only the skin, but also the inner epidermis in tomato fruit [57], and this distribution was very similar to GAX. GAX is present in the vascular bundle of dicotyledons and contributes to cell wall firmness. Therefore, GAX was suggested to be involved in fruit tissue firmness in the inner epidermis and in fruit shape in both the inner and outer cuticle. Changes in the cell wall network caused by biosynthesis of XG and GAX and tissue-specific distribution of these polysaccharides during fruit ripening is important for the regulation of fruit softening and maintenance of the globular form of tomato fruits.

## Supporting Information

File S1 Figure S1. Comparison of fresh weight-based and protein-based β-xylosidase activities in fruit tissues. Comparison of fresh weight-based (A) and protein-based (B) β-xylosidase activity data. Total cell wall protein was extracted from each fruit tissue and assayed for β-xylosidase activity. The five tissues analyzed were skin, mesocarp/endocarp, septum, locular tissue, and seeds from the overripe-stage fruit. Figure S2. Changes in glucose content differed among fruit tissues during ripening. Glucose content per 1-g fresh weight of each fruit tissue. The four tissues analyzed were: skin, mesocarp/endocarp, septum, and locular tissue. Ripening stage: I, immature green; M, mature green; B, breaker; T, turning; R, red ripe; O, overripe. ± SD of three independent replicates.
(DOCX)
