# Supplementary material for: Regulatory Specialization of Xyloglucan (XG) and Glucuronoarabinoxylan (GAX) in Pericarp Cell Walls during Fruit Ripening in Tomato (Solanum lycopersicum)
Source: PLoS One. 2014 Feb 26;9(2):e89871. doi: 10.1371/journal.pone.0089871 (PMC3935947; doi:10.1371/journal.pone.0089871)
Supplement: File S1 — Figure S1. Comparison of fresh weight-based and protein-based β-xylosidase activities in fruit tissues. Comparison of fresh weight-based (A) and protein-based (B) β-xylosidase activity data. Total cell wall protein was extracted from each fruit tissue and assayed for β-xylosidase activity. The five tissues analyzed were skin, mesocarp/endocarp, septum, locular tissue, and seeds from the overripe-stage fruit. Figure S2. Changes in glucose content differed among fruit tissues during ripening. Glucose content per 1-g fresh weight of each fruit tissue. The four tissues analyzed were: skin, mesocarp/endocarp, septum, and locular tissue. Ripening stage: I, immature green; M, mature green; B, breaker; T, turning; R, red ripe; O, overripe. ± SD of three independent replicates. (DOCX) [file pone.0089871.s001.docx]

**Supporting Information legends**

Figure S1. Comparison of the fresh weight-based and the protein-based β-Xylosidase activities in fruit tissues.

**
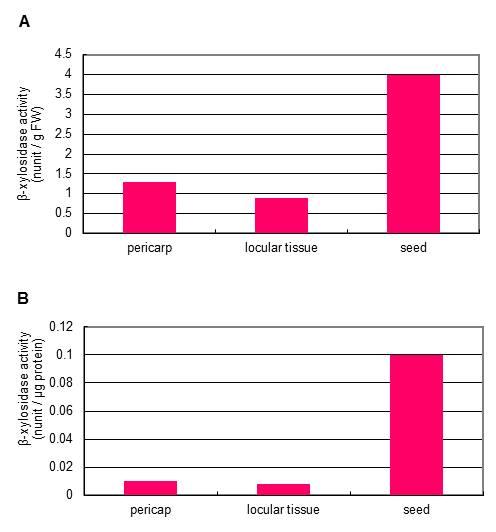
**

Figure S2. Changes in glucose content differed in fruit tissues during ripening.

**
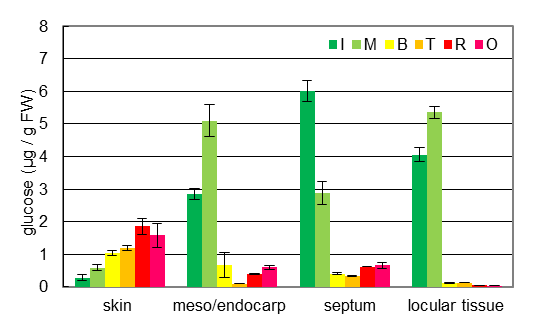
**
